# Progress Toward Regional Measles Elimination — Worldwide, 2000–2017

**DOI:** 10.15585/mmwr.mm6747a6

**Published:** 2018-11-30

**Authors:** Alya Dabbagh, Rebecca L. Laws, Claudia Steulet, Laure Dumolard, Mick N. Mulders, Katrina Kretsinger, James P. Alexander, Paul A. Rota, James L. Goodson

**Affiliations:** ^1^Department of Immunization, Vaccines, and Biologicals, World Health Organization, Geneva, Switzerland; ^2^Global Immunization Division, Center for Global Health, CDC; ^3^Division of Viral Diseases, National Center for Immunization and Respiratory Diseases, CDC.

In 2010, the World Health Assembly set three milestones for measles prevention to be achieved by 2015: 1) increase routine coverage with the first dose of measles-containing vaccine (MCV1) among children aged 1 year to ≥90% at the national level and to ≥80% in every district; 2) reduce global annual measles incidence to less than five cases per million population; and 3) reduce global measles mortality by 95% from the 2000 estimate (*1*).* In 2012, the World Health Assembly endorsed the Global Vaccine Action Plan (GVAP),^†^ with the objective of eliminating measles^§^ in four of the six World Health Organization (WHO) regions by 2015 and in five regions by 2020. Countries in all six WHO regions have adopted goals for measles elimination by 2020. This report describes progress toward global measles control milestones and regional measles elimination goals during 2000–2017 and updates a previous report (*2*). During 2000–2017, estimated MCV1 coverage increased globally from 72% to 85%; annual reported measles incidence decreased 83%, from 145 to 25 cases per million population; and annual estimated measles deaths decreased 80%, from 545,174 to 109,638. During this period, measles vaccination prevented an estimated 21.1 million deaths. However, measles elimination milestones have not been met, and three regions are experiencing a large measles resurgence. To make further progress, case-based surveillance needs to be strengthened, and coverage with MCV1 and the second dose of measles-containing vaccine (MCV2) needs to increase; in addition, it will be important to maintain political commitment and ensure substantial, sustained investments to achieve global and regional measles elimination goals.

## Immunization Activities

WHO and the United Nations Children’s Fund (UNICEF) use data from administrative records and vaccination coverage surveys reported annually by 194 countries to estimate coverage with MCV1 and MCV2 delivered through routine immunization services.^¶^ During 2000–2017, estimated MCV1 coverage increased globally from 72% to 85% (Table 1), although coverage has remained 84%–85% since 2010, and considerable variation in regional coverage exists. Since 2013, MCV1 coverage has remained relatively constant in the African Region (AFR) (69%–70%), the Region of the Americas (AMR) (92%), the European Region (EUR) (93%–95%), and the Western Pacific Region (WPR) (96%–97%). During 2013–2017, MCV1 coverage increased from 78% to 81% in the Eastern Mediterranean Region (EMR) and from 84% to 87% in the South-East Asia Region (SEAR). WPR is the only region to achieve and sustain >95% MCV1 coverage since 2006. Among the 73 countries that receive funding through Gavi, the Vaccine Alliance (Gavi-eligible countries),** MCV1 coverage increased during 2000–2017, from 59% to 79% (Table 1). Globally, 118 (61%) countries achieved ≥90% MCV1 coverage in 2017, an increase from 85 (44%) countries in 2000, and a slight decrease from 120 (62%) countries in 2016. During 2000–2017, the largest increases in the percentage of countries with ≥90% MCV1 coverage were in AFR (from 9% to 34%) and SEAR (from 27% to 64%); among Gavi-eligible countries, the percentage of countries with ≥90% MCV1 coverage increased from 15% to 44% (Table 1). In 2017, 78 (40%) countries reached ≥95% MCV1 coverage nationally, and 45 (23%) countries achieved ≥80% MCV1 coverage in all districts. Globally, an estimated 20.8 million infants did not receive MCV1 through routine immunization services in 2017. The six countries with the most unvaccinated infants were Nigeria (3.9 million), India (2.9 million), Pakistan (1.2 million), Indonesia (1.2 million), Ethiopia (1.1 million), and Angola (0.7 million).

**TABLE 1:** Estimates of coverage with the first and second doses of measles-containing vaccine administered through routine immunization services, reported measles cases and incidence, estimated measles deaths,* and estimated measles deaths averted by vaccination by World Health Organization (WHO) region — worldwide, 2000 and 2017

WHO region or Gavi-eligible countries (no. of countries in category)/Year	MCV1^†^ coverage, %	Countries with ≥90% MCV1 coverage, %	MCV2^†^ coverage, %	Reporting countries with <5 measles cases/million, %	Reported measles cases,^§^ no.	Measles incidence^§,¶^	Estimated no. of measles deaths (95% CI)	Estimated mortality reduction, 2000–2017, %	Cumulative measles deaths averted by vaccination, 2000–2017, no.
**African (47)**									
**2000**	53	9	5	8	520,102	835	348,207 (239,261–565,071)	86	10,402,672
**2017**	70	34	25	53	72,603	69	48,017 (22,167–166,341)		
**Americas (35)**									
**2000**	93	63	43	89	1,754	2.1	NA	—	92,777
**2017**	92	63	74	97	775	1.7	NA		
**Eastern Mediterranean (21)**									
**2000**	72	57	29	17	38,592	90	42,977 (23,351–77,054)	43	2,535,740
**2017**	81	62	67	55	36,427	57	24,321 (2,418–70,806)		
**European (53)**									
**2000**	91	60	48	45	37,421	50	346 (109–1,801)	71	90,134
**2017**	95	83	90	57	24,356	27	100 (1–1,356)		
**South-East Asia (11)**									
**2000**	63	27	3	0	78,558	51	143,333 (100,362–203,472)	75	6,699,720
**2017**	87	64	77	45	28,474	14	35,925 (21,401–83,156)		
**Western Pacific (27)**									
**2000**	85	48	2	30	177,052	105	10,311 (5,153–65,828)	88	1,230,932
**2017**	97	59	94	80	10,695	6	1,275 (136–54,960)		
**Total (194)**									
**2000**	**72**	**44**	**15**	**38**	**853,479**	**145**	**545,174 (368,236–913,226)**	**80**	**21,051,974**
**2017**	**85**	**61**	**67**	**65**	**173,330**	**25**	**109,638 (46,123–376,619)**		
Gavi-eligible countries (73)**									
**2000**	59	15	2	14	645,880	258	536,122 (364,323–839,659)	80	19,320,191
**2017**	79	44	51	58	138,334	40	107,232 (45,839–314,724)		

**Abbreviations:** CI = confidence interval; Gavi = Gavi, the Vaccine Alliance; MCV1 = first dose of measles-containing vaccine; MCV2 = second dose of measles-containing vaccine; NA = not applicable; UNICEF = United Nations Children’s Fund.

* Mortality estimates for 2000 might be different from previous reports. When the model used to generate estimated measles deaths is rerun each year using the new WHO/UNICEF Estimates of National Immunization Coverage data, as well as updated surveillance data, adjusted results for each year, including the baseline year, are also produced and updated.

^†^ Coverage data: WHO/UNICEF Estimates of National Immunization Coverage, July 15, 2018 update. http://www.who.int/immunization/monitoring_surveillance/data/en.

^§^ Reported case data: measles cases (2017) from World Health Organization, as of July 15, 2018 (http://apps.who.int/immunization_monitoring/globalsummary/timeseries/tsincidencemeasles.html). Reported cases are a sizeable underestimate of the actual number of cases, accounting for the inconsistency between reported cases and estimated deaths.

^¶^ Cases per 1 million population; population data from United Nations, Department of Economic and Social Affairs, Population Division, 2017. Any country not reporting data on measles cases for that year was removed from both the numerator and denominator.

** Gavi, the Vaccine Alliance (Gavi), previously known as the Global Alliance for Vaccines and Immunization (GAVI), is a public-private global health partnership committed to increasing access to immunization in poor countries. Gavi-eligible countries are those that received funding support from Gavi, the Vaccine Alliance. Countries are eligible to apply for Gavi support when their Gross National Income (GNI) per capita is ≤US$1,580 on average over the past three years (according to World Bank data published every year on July 1). In Gavi phase I (2000 to 2006), the GNI per capita eligibility threshold was US$1,000 (based on 1998 World Bank data). In Gavi phase II (2007 to 2010), country eligibility was based on the World Bank GNI per capita data for 2003. The eligibility threshold was maintained at the initial level of US$1,000. Since January 1, 2011, Gavi phase III, the threshold is adjusted for inflation annually. All 73 Gavi-eligible countries are included here, even if they graduated from Gavi support during 2000–2017. Timor Leste and South Sudan data were not available for the year 2000.

Estimated MCV2 coverage increased globally from 15% in 2000 to 67% in 2017, largely because of an increase in the number of countries providing MCV2 nationally from 98 (51%) in 2000 to 167 (86%) in 2017 (Table 1). Three countries introduced MCV2 in 2017 (Laos, Namibia, and Nicaragua). During 2000–2017, the largest increases in regional MCV2 coverage were from 3% to 77% in SEAR, and from 2% to 94% in WPR. Among Gavi-eligible countries, MCV2 coverage increased from 2% to 51% during 2000–2017.

During 2017, approximately 205 million persons received supplementary doses of measles-containing vaccine (MCV) during 53 supplementary immunization activities (SIAs)^††^ implemented in 39 countries (Table 2). Based on doses administered, SIA coverage was ≥95% in 26 (49%) SIAs. During 2010–2017, a total of 1,476,826,523 persons were vaccinated globally through 443 measles SIAs (an average of 55 SIAs per year); 172 (39%) SIAs included at least one other health intervention.

**TABLE 2:** Measles supplementary immunization activities (SIAs)* and the delivery of other child health interventions, by World Health Organization (WHO) region and country — African, Eastern Mediterranean, European, South-East Asia, and Western Pacific Regions, 2017

WHO region/country	Age group targeted	Extent of SIA	No. of children (%) reached in targeted age group^†^	% coverage based on survey results	Other interventions delivered
**African**					
Algeria	6–14 yrs	N	3,154,279 (45)	—	Rubella vaccine
Burundi	9 mos–14 yrs	N	4,126,421 (99)	98	Rubella vaccine
Central African Republic	6 mos–14 yrs	SN	28,155 (98)	—	—
Central African Republic	6 mos–14 yrs	SN	63,823 (131)	—	Vitamin A, deworming
Chad	9–59 mos	SN	707,103 (102)	—	—
Democratic Republic of the Congo	6–59 mos	SN	5,466,923 (103)	89	—
Ethiopia	9 mos–14 yrs	SN	21,225,199 (96)	93	—
Ethiopia	6–179 mos	SN	2,524,841 (98)	—	—
Gabon	9–59 mos	N	200,648 (75)	—	Vitamin A, bOPV
Guinea	6–10 yrs	SN	148,344 (104)	—	—
Guinea	6–10 yrs	SN	662,733 (96)	—	—
Guinea	6–59 mos	SN	1,315,918 (104)	—	—
Lesotho	9 mos–14 yrs	N	540,017 (89)	92	Rubella vaccine, vitamin A, bOPV, deworming
Malawi	9 mos–14 yrs	N	8,132,788 (102)	93	Rubella vaccine, vitamin A, deworming
Nigeria	9–59 mos	N	40,044,875 (107)	88	—
Rwanda	9–15 yrs	SN	93,893 (98)	—	Rubella vaccine
Rwanda	9–59 mos	N	1,508,834 (102)	97	Rubella vaccine, vitamin A, deworming
Senegal	9–59 mos	N	2,226,482 (107)	91	Rubella vaccine
South Africa	6–59 mos	N	4,255,588 (80)	—	—
South Africa	5–14 yrs	SN	846,642 (82)	—	—
South Sudan	6–59 mos	N	1,950,955 (84)	—	Vitamin A, OPV, deworming
**Eastern Mediterranean**					
Afghanistan	9–59 mos	SN	1,053,452 (97)	—	—
Djibouti	4–8 yrs	N	11,628 (92)	—	Vitamin A, deworming
Iraq	6–13 yrs	SN	319,314 (82)	—	Rubella vaccine, mumps vaccine
Kuwait	1–19 yrs	N	165,296 (16)	—	Rubella vaccine, mumps vaccine
Lebanon	1–15 yrs	SN	1,938 (83)	—	Rubella vaccine, mumps vaccine, OPV, IPV, PCV
Libya	3–6 yrs	N	721,488 (101)	—	Rubella vaccine, mumps vaccine
Oman	20–35 yrs	N	1,658,642 (92)	—	Rubella vaccine, mumps vaccine
Yemen	6 mos–15 yrs	SN	205,731 (41)	—	Rubella vaccine
Yemen	6 mos–15 yrs	SN	166,654 (100)	—	Rubella vaccine
**Europe**					
Cyprus	14 yrs	N	6,176 (86)	—	Rubella vaccine, mumps vaccine
Cyprus	6–12 yrs	N	7,446 (92)	—	Rubella vaccine, mumps vaccine
Cyprus	6–12 yrs	N	7,957 (91)	—	Rubella vaccine, mumps vaccine
Georgia	6–30 yrs	N	7,501 (15)	—	Rubella vaccine, mumps vaccine
Romania	9–11 mos	N	97,958 (30)	—	Rubella vaccine, mumps vaccine
Tajikistan	1–9 yrs	N	1,938,190 (100)	—	Rubella vaccine
Turkey	refugees	N	85,670 (21)	—	Rubella vaccine, mumps vaccine, Hepatitis B vaccine, DTaP vaccine, IPV, Hib vaccine
Turkey	refugees	N	28,908 (7)	—	Rubella vaccine, mumps vaccine
Turkey	refugees	N	28,732 (7)	—	Rubella vaccine, mumps vaccine
Ukraine	1–9 yrs	N	163,782 (57)	—	Rubella vaccine, mumps vaccine
Ukraine	6–9 yrs	N	154,430 (67)	—	Rubella vaccine, mumps vaccine
**South-East Asia**					
Bangladesh	9 mos–<5 yrs	SN	1,552,374 (100)	—	Rubella vaccine
Bangladesh	6 mos–<15 yrs	SN	490,501 (107)	—	Rubella vaccine, OPV
Bhutan	9 mos– 40 yrs	N	263,337 (98)	—	Rubella vaccine
India^§^	9 mos–15 yrs	N	59,156,720 (98)	—	Rubella vaccine
Indonesia	9 mos–15 yrs	SN	35,307,148 (101)	—	Rubella vaccine
Maldives	15–25 yrs	N	46,835 (76)	—	Rubella vaccine
Maldives	8–14 yrs	N	1,645 (77)	—	Rubella vaccine
**Western Pacific**					
Cambodia	6–59 mos	N	1,452,821 (90)	75	Rubella vaccine
Fiji	12 mos–11 yrs	N	178,069 (95)	—	Rubella vaccine
Laos	9 mos–<5 yrs	N	703,924 (100)	—	Rubella vaccine, bOPV
Federated States of Micronesia	12–60 mos	SN	1,491(79)	—	Rubella vaccine, mumps vaccine
Samoa	1–12 yrs	N	57,229 (95)	—	Rubella vaccine

**Abbreviations:** bOPV = bivalent oral poliovirus vaccine; DPT = diphtheria and pertussis toxoids and tetanus vaccine; DT = diphtheria and tetanus toxoids; DTaP = diphtheria and tetanus toxoids and acellular pertussis vaccine; Hib = *Haemophilus influenzae* type b vaccine; IPV = inactivated polio vaccine; N = national; OPV = oral poliovirus vaccine; PCV = pneumococcal conjugate vaccine; Penta = pentavalent (DTP, hepatitis B, Hib) vaccine; SIA = supplementary immunization activity; SN = subnational.

* SIAs generally are carried out using two approaches: 1) An initial, nationwide catch-up SIA targets all children aged 9 months to 14 years; it has the goal of eliminating susceptibility to measles in the general population. Periodic follow-up SIAs then target all children born since the last SIA. 2) Follow-up SIAs are generally conducted nationwide every 2–4 years and target children aged 9–59 months; their goal is to eliminate any measles susceptibility that has developed in recent birth cohorts and to protect children who did not respond to the first measles vaccination. The exact age range for follow-up SIAs depends on the age-specific incidence of measles, coverage with 1 dose of measles-containing vaccine, and the time since the last SIA.

^†^ Values >100% indicate that the number of doses administered exceeded the estimated target population.

^§^ Rollover national campaigns started the previous year or will continue into the next year.

## Reported Measles Incidence

In 2017, 189 (97%) countries conducted measles case-based surveillance in at least part of the country, and 191 (98%) had access to standardized quality-controlled testing through the WHO Global Measles and Rubella Laboratory Network. However, surveillance was weak in many countries, and fewer than half of the countries reporting surveillance indicators (73 of 152; 48%) achieved the sensitivity indicator target of two or more discarded measles and rubella^§§^ cases per 100,000 population.

Countries report the aggregate number of incident measles cases^¶¶^^,^*** to WHO and UNICEF annually through the Joint Reporting Form.^†††^ During 2000–2017, the number of measles cases reported worldwide decreased 80%, from 853,479 in 2000 to 173,330 in 2017, and measles incidence decreased 83%, from 145 to 25 cases per million population (Table 1). Compared with the reported number of cases (132,328) and incidence (19 cases per million) in 2016, both cases and incidence increased in 2017, in part because eight more countries reported case data in 2017 (184 of 194; 95%) than did in 2016 (176 of 194; 91%).^§§§^ The percentage of reporting countries with annual measles incidence of <5 cases per million population increased from 38% (64 of 169) in 2000 to 69% (122 of 176) in 2016, and then decreased to 65% (119 of 184) in 2017. During 2016–2017, reported measles cases increased 31% globally, 100% in AFR, 6,358% in AMR, 481% in EMR, 458% in EUR, and 3% in SEAR, but decreased 82% in WPR. In Gavi-eligible countries, reported cases increased 45% from 2016.

Genotypes of viruses isolated from measles cases were reported by 76 (59%) of the 129 countries that reported at least one measles case in 2017. Among the 24 recognized measles virus genotypes, 11 were detected during 2005–2008, eight during 2009–2014, six in 2015, and five in 2016 and 2017, excluding those from vaccine reactions and cases of subacute sclerosing panencephalitis, a fatal progressive neurologic disease caused by persistent measles virus infection (*3*).^¶¶¶^ In 2017, among 5,789 reported measles virus sequences,**** 2,641 (45.6%) were genotype B3 (53 countries); 15 (0.26%) were D4 (two countries); 2,542 (43.9%) were D8 (49 countries); 46 (0.80%) were D9 (six countries); and 545 (9.4%) were H1 (11 countries).

## Measles Mortality Estimates

A previously described model for estimating measles disease and mortality was updated with new measles vaccination coverage data, case data, and United Nations population estimates for all countries during 2000–2017, enabling derivation of a new series of disease and mortality estimates. For countries with previously anomalous estimates, the model was modified slightly to generate mortality estimates consistent with the observed case data (*4*). Based on the updated data, the estimated number of measles cases declined from 28,493,539 (95% confidence interval [CI] = 19,808,871–64,780,514) in 2000 to 6,732,904 (CI = 2,950,042–36,842,865) in 2017. During this period, estimated measles deaths decreased 80%, from 545,174 (CI = 368,236–913,226) in 2000 to 109,638 (CI = 46,123–376,619) in 2017 (Table 1). During 2000–2017, compared with no measles vaccination, measles vaccination prevented an estimated 21.1 million deaths globally and 19.3 million deaths among Gavi-eligible countries (Figure) (Table 1).

**FIGURE:**
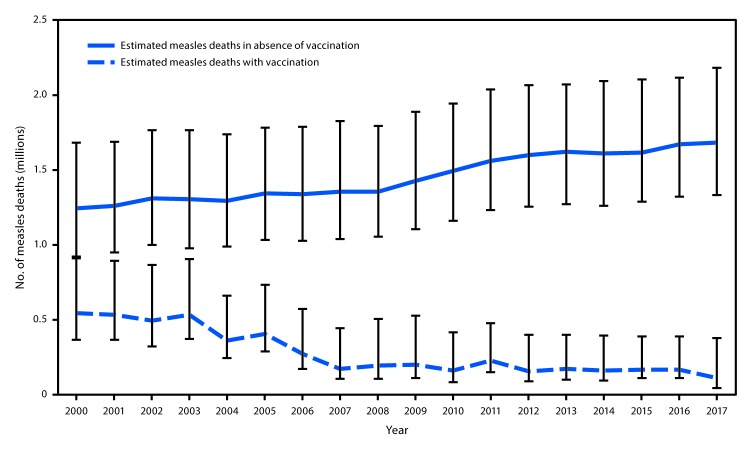
Estimated annual number of measles deaths with and without vaccination programs — worldwide, 2000–2017* * Deaths prevented by vaccination are indicated by the area between estimated deaths with vaccination and those without vaccination (cumulative total of 21.1 million deaths prevented during 2000–2017). Error bars represent upper and lower 95% confidence limits around the point estimate.

## Regional Verification of Measles Elimination

In 2017, AFR and EMR established regional verification commissions (RVCs); thus, all six regions now have RVCs. In September 2016, the AMR RVC declared the region free of endemic measles (*5*). In 2017, the EUR RVC verified measles elimination in 37 (70%) countries and the reestablishment of endemic measles virus transmission in the Russian Federation and in Germany (*6*). In SEAR, Maldives and Bhutan were verified as having eliminated measles in 2017 (*7*). In WPR, six (22%) countries (Australia, Brunei, Cambodia, Japan, New Zealand, and South Korea) and two areas, Hong Kong Special Autonomous Region (China) and Macao Special Autonomous Region (China), had verified measles elimination in 2017 (*8*). No EMR or AFR countries had verified elimination in 2017.

## Discussion

During 2000–2017, increased coverage with MCV administered through routine immunization programs and SIAs, and other global measles elimination efforts contributed to an 83% decrease in reported measles incidence and an 80% reduction in estimated measles mortality. Measles vaccination prevented an estimated 21.1 million deaths during this period; the large majority of deaths averted were in AFR and among Gavi-eligible countries. Global MCV2 coverage has steadily increased since 2000; in 2017, 167 (86%) countries provided MCV2. In 2017, MCV1 and MCV2 coverage in WPR was ≥94%, and measles incidence in this region was at an all-time low. The increasing number of countries verified as having achieved measles elimination indicates progress toward global interruption of measles virus transmission.

Despite this progress, however, the 2015 global milestones have not been achieved; global MCV1 coverage has stagnated for nearly a decade; global MCV2 coverage is only at 67% despite steady increases; and SIA quality was inadequate to achieve ≥95% coverage in several countries. Since 2016, measles incidence has increased globally and in five of the six WHO regions. Furthermore, as of July 2018, endemic measles has been reestablished in Venezuela because of the sustained transmission of measles virus for >12 months; the remaining 34 AMR countries continue to maintain their measles elimination status, but the ongoing outbreak in Venezuela has led to measles virus importations and outbreaks in bordering AMR countries. In addition, the measles resurgence in Europe has likely led to reestablished endemic measles in some EUR countries. These outbreaks highlight the fragility of gains made toward global and regional measles elimination goals. Continuing to increase MCV1 and MCV2 coverage is critical to both the achievement and sustainability of the global and regional measles elimination goals. Meanwhile, conducting high quality SIAs that reach unvaccinated and undervaccinated children will prevent future outbreaks that are costly in terms of morbidity and mortality and are disruptive to immunizations service delivery. It is important to have high-performing surveillance for early detection of outbreaks; and when outbreaks do occur, thorough outbreak investigations are needed to better understand and address the underlying causes of the outbreak and why children are being missed by immunization delivery systems.

The findings in this report are subject to at least three limitations. First, SIA administrative coverage data might be biased by inaccurate reports of the number of doses delivered, doses administered to children outside the target age group, and inaccurate estimates of the target population size. Second, large differences between the estimated and reported incidence indicate variable surveillance sensitivity, making comparisons between countries and regions difficult to interpret. Finally, the accuracy of estimates from the measles mortality model is affected by biases in all model inputs, including country-specific measles vaccination coverage and measles case-based surveillance data.

Monitoring progress toward measles elimination goals could be improved by establishing updated indicators. For example, the WHO Strategic Advisory Group of Experts on Immunization recently approved country classifications, and updates to the framework for the verification of measles elimination will standardize monitoring of countries’ progress toward verified elimination (*9*). Moreover, synergizing future global health efforts and capitalizing on immunization partners’ investments could be enhanced by dovetailing measles and rubella elimination strategies with post-GVAP immunization targets and strategies.

Strengthening routine immunization and continuing to conduct high-quality SIAs will help achieve global and regional measles elimination goals, improve overall vaccination coverage and equity, and assist in attaining universal health coverage. It is important that countries continue to strengthen case-based surveillance and increase MCV1 and MCV2 coverage and that immunization partners continue to raise the visibility of measles elimination goals and secure political commitment to these goals and sustained investments in health systems.

**Summary**

**What is already known about this topic?**

In 2012, the World Health Assembly endorsed the Global Vaccine Action Plan; as a result, countries in all six World Health Organization regions have adopted goals for elimination of measles by 2020.

**What is added by this report?**

During 2000–2017, annual reported measles incidence decreased 83%, and annual estimated measles deaths decreased 80%. Since 2000, global measles elimination efforts have prevented an estimated 21.1 million deaths. However, measles elimination milestones have not been met, and three regions are experiencing a large measles resurgence.

**What are the implications for public health practice?**

To make further progress, case-based surveillance needs to be strengthened, and coverage with the first and second dose of measles-containing vaccine needs to increase; moreover, it is important to maintain political commitment, and secure substantial, sustained investments.

## Footnotes

† The Global Vaccine Action Plan is the implementation plan of the Decade of Vaccines, a collaboration between WHO; UNICEF; the Bill and Melinda Gates Foundation; the National Institute of Allergy and Infectious Diseases; the African Leaders Malaria Alliance; Gavi, the Vaccine Alliance; and others to extend the full benefit of immunization to all persons by 2020 and beyond. In addition to 2015 targets, it also set a target for measles and rubella elimination in five of the six WHO regions by 2020. http://www.who.int/immunization/global_vaccine_action_plan/en; http://apps.who.int/gb/ebwha/pdf_files/wha65/a65_22-en.pdf.

§ Measles elimination is defined as the absence of endemic measles virus transmission in a region or other defined geographic area for ≥12 months, in the presence of a high quality surveillance system that meets targets of key performance indicators.

¶ For MCV1, among children aged 1 year or, if MCV1 is given at age ≥1 year, among children aged 24 months. For MCV2, among children at the recommended age for administration of MCV2, per the national immunization schedule. WHO/UNICEF estimates of national immunization coverage are available at http://www.who.int/immunization/monitoring_surveillance/data/en.

** Gavi, the Vaccine Alliance (Gavi), previously known as the Global Alliance for Vaccines and Immunization (GAVI), is a public-private global health partnership committed to increasing access to immunization in poor countries. Gavi-eligible countries are those that received funding support from Gavi. Countries are eligible to apply for Gavi support when their Gross National Income (GNI) per capita is ≤US$1,580 on average over the past 3 years (according to World Bank data published every year on July 1). In Gavi phase I (2000 to 2006), the GNI per capita eligibility threshold was US$1,000 (based on 1998 World Bank data). In Gavi phase II (2007 to 2010), country eligibility was based on the World Bank GNI per capita data for 2003. The eligibility threshold was maintained at the initial level of US$1,000. Since January 1, 2011, Gavi phase III, the threshold is adjusted for inflation annually. All 73 Gavi-eligible countries are included here, even if they graduated from Gavi support during 2000–2017. Timor Leste and South Sudan data were not available for the year 2000.

†† Supplemental immunization activities (SIAs) generally are carried out using two target age ranges. An initial, nationwide catch-up SIA focuses on all children aged 9 months–14 years, with the goal of eliminating susceptibility to measles in the general population. Periodic follow-up SIAs then focus on all children born since the last SIA. Follow-up SIAs generally are conducted nationwide every 2–4 years and focus on children aged 9–59 months; their goal is to eliminate any measles susceptibility that has developed in recent birth cohorts and to protect children who did not respond to MCV1.

§§ A discarded case is defined as a suspected case that has been investigated and discarded as nonmeasles and as nonrubella using 1) laboratory testing in a proficient laboratory or 2) epidemiological linkage to a laboratory-confirmed outbreak of a communicable disease that is not measles or rubella. The discarded case rate is used to measure the sensitivity of measles surveillance.

¶¶
http://apps.who.int/immunization_monitoring/globalsummary/timeseries/tsincidencemeasles.html .

*** Measles cases are defined differently in different countries. Some countries define measles cases as those that are laboratory-confirmed or epidemiologically linked; others define measles cases as those that are laboratory-confirmed, epidemiologically linked, or clinically compatible. Laboratory-confirmed cases are suspected measles cases with specimens that have detectable measles virus–specific immunoglobulin class M antibodies, or specimens from which measles virus can be isolated or measles virus genome can be detected in appropriate clinical specimens by a proficient laboratory. Epidemiologically linked confirmed measles cases are suspected measles cases that have not been confirmed by a laboratory but are geographically and temporally related to a laboratory-confirmed case or, in the event of a chain of transmission, to another epidemiologically confirmed measles case, with dates of rash onset between cases occurring 7–21 days apart. Clinically compatible measles cases are suspected measles cases with fever and maculopapular rash and cough, coryza, or conjunctivitis, for which no adequate clinical specimen was collected and which have not been linked epidemiologically to a laboratory-confirmed case of measles or to a laboratory-confirmed case of another communicable disease.

†††
http://www.who.int/immunization/monitoring_surveillance/routine/reporting/en/ .

§§§ Ten countries did not report measles case data in 2017: Brazil, Cook Islands, Fiji, Marshall Islands, Morocco, Nauru, Niue, Tuvalu, United States of America, and Vanuatu. Eighteen countries did not report case data in 2016: Belgium, Cabo Verde, Cook Islands, Haiti, Ireland, Italy, Kiribati, Marshall Islands, Monaco, Morocco, Mozambique, Niue, Samoa, Singapore, Suriname, Tuvalu, United States of America, and Vanuatu.

¶¶¶ http://dx.doi.org/10.1016/B978-0-444-53488-0.00027-4.

**** Sequences were for the 450 nucleotides coding for the carboxy-terminal 150 amino acids of the nucleoprotein of measles virus. Data (as of October 4, 2018) were available from the Measles Nucleotide Surveillance database. http://www.who-measles.org/Public/Web_Front/main.php.
